# Phacoemulsification in the anterior chamber: An alternative surgical technique in post-vitrectomy cataract

**DOI:** 10.12669/pjms.346.15962

**Published:** 2018

**Authors:** Tao Yu, Xu-Guang Han, Ying-Mei Li, Yu-Guang Zhang

**Affiliations:** 1 *Tao Yu, Qianfoshan Hospital Affiliated to Shandong University, Jinan, 250014, Shandong, China*; 2 *Xu-Guang Han, Jinan Aier Ophthalmology Hospital, Aier Eye Hospital Group, Jinan 250014, Shandong, China*; 3 *Ying-Mei Li, Jinan Second People’s Hospital, Jinan Eye Hospital, Jinan Eye Institute, Jinan 250012, Shandong, China*; 4 *Yu-Guang Zhang, Jinan Second People’s Hospital, Jinan Eye Hospital, Jinan Eye Institute, Jinan 250012, Shandong, China*

**Keywords:** Anterior chamber, Cataract, Phacoemulsification, Post-vitrectomy

## Abstract

**Objective:**

To evaluate the characteristics, safety and effectiveness of a modified technique of phacoemulsification in post-vitrectomy cataracts.

**Methods:**

This retrospective clinical trial comprised 31 patients (31 eyes) with post-vitrectomy cataract, who had undergone phacoemulsification combined with intraocular lens implantation. An alternative surgical technique known as phacoemulsification in the anterior chamber was used for nucleus management in those cases. The following parameters were evaluated: best corrected visual acuity (BCVA), ocular inflammation, intraocular pressure, endothelial cell count and surgical complications.

**Results:**

Three months after surgery, the BCVA improved significantly compared with that before surgery (*Z*=-10.547; *p*<0.05). There were no significant differences in IOP before and after surgery (*Z*=-0.474; *p*>0.05). There was a statistically significant postoperative decrease in endothelial cell densities (*Z*=-3.916; *p*<0.05). The mean endothelial cell loss was -8.71%. A little inflammatory response in the anterior chamber in 11 eyes and mild corneal edema in 8 eyes were observed on the first day after surgery, which subsided after a week. The posterior capsular opacification were observed in three eyes, two of which were denser, and the YAG laser was performed for posterior capsular incision. No obvious surgical complications occurred.

**Conclusion:**

The modified technique of phacoemulsification, with phacoemulsification in the anterior chamber, is safe and effective to deal with post-vitrectomy cataracts.

## INTRODUCTION

With the advancement of technology, modern vitrectomy has greatly improved the prognosis of patients with vitreous retinopathy. However, the occurrence of post-vitrectomy cataracts can affect the patient’s vision, as well as the observation and treatment of the fundus.^1^ The preferred method of surgery to correct post-vitrectomy cataract is phacoemulsification and intraocular lens implantation.^2^,^3^

The conventional method of this surgery is to perform phacoemulsification inside the lens capsular bag.^2^ This surgery itself has many challenging features caused by the intraocular structural changes after vitrectomy, such as the small pupil, flabby suspensory ligament, fragile posterior capsule, and more mobility of the posterior capsule, which increase the difficulty and risk of the surgery.^4^,^5^ In this study, we improved the traditional technique of nuclear emulsification by performing phacoemulsification outside the capsule in order to minimize surgical difficulty and help reduce complications.

## METHODS

### Subjects

We retrospectively analyzed 31 patients (31 eyes) with post-vitrectomy cataract from January 2016 to May 2017 in Jinan Second People’s Hospital, Jinan Eye Hospital. The time after vitrectomy was five months to two years, with an average of 10 months. The primary disease of vitrectomy was as follows: 10 eyes of rhegmatogenous retinal detachment retinal detachment, five eyes of macular hole, seven eyes of vitreous hemorrhage caused by retinal vein occlusion, nine eyes of proliferative diabetic retinopathy. In these cases, 12 eyes had been filled with silicone oil and nine eyes had been filled with gas, which had been removed before the cataract surgery. Preoperative conventional slit lamp microscopy showed the different types of cataracts: eight eyes were cortical cataract, 14 eyes were nuclear cataract, and nine eyes were posterior subcapsular cataract. All surgery was performed by the same experienced surgeon. The study protocol was approved by the Ethics Committee of Jinan Second People’s Hospital, Jinan Eye Hospital and adhered to the tenets of the Declaration of Helsinki.

The exclusion criteria were previous corneal pathology (dystrophic or degenerative such as Fuchs endothelial dystrophy or advanced trachoma); pseudoexfoliation syndrome; history of the other intraocular surgery, glaucoma, ocular hypertension, anterior uveitis; anterior chamber depth (ACD) less than 2.5 mm, ocular axial length less than 21.0 mm, corneal endothelial cell density less than 2000 cells/mm^2^, retina and choroid detachment shown by B ultrasound.

### Surgical steps

The operation was performed under the surface anesthesia. Both the phaco and side port incisions were made on the clear cornea. After injecting viscoelastic agent, continuous circular capsulorhexis was performed. Hydrodissection must be done gentlely and sufficiently to ensure that the crystal nucleus may be adequately rotated. The nucleus was rotated out of the capsular bag by using an iris restorer or a viscoelastic needle. The phaco-chop phacoemulsification techniques were utilized for nuclear management in the anterior chamber. All cases underwent posterior capsular polishing, and posterior capsulorhexis was performed for cases with severe opacity.^6^ The intraocular lens was placed in the capsular bag, and the phaco and sideport incisions were closed.

### Observation projects

Postoperative follow-up observation was performed for 3 to 12 months. The preoperative and postoperative observation parameters included: the best corrected visual acuity, corneal edema, anterior chamber inflammation, intraocular pressure, corneal endothelium cell density and surgical complications.

### Statistical Analysis

The preoperative and postoperative examination parameters were selected for analysis using the Statistical Package for the Social Sciences version 17.0 (SPSS Inc, Chicago, III). The Mann-Whitney U test was performed to compare the BCVA (logMAR). The *χ*^2^ were performed to compare the other parameters. A *p-*values less than 0.05 was considered to be statistically significant difference.

## RESULTS

All operations were completed successfully. In 10 eyes, the pupil did not dilate properly and a step-wise approach was utilized to small pupil anagement (Posterior synechiolysis, viscomydriasis, pupillary membrane dissection, and stretch pupilloplasty). The posterior capsular fibrosis or plaques were found in 11 eyes. Among them, the plaques had been removed by posterior capsular polishing in six eyes, but the very dense plaques had been managed by posterior capsulorhexis in the other eyes. There were varying degrees of anterior chamber depth and pupil size changes during the operation. There were no serious complications during the operation, such as sclera collapse, nucleus sinking into vitreous chamber and bleeding in epichoroidal space.

On the first day after cataract surgery, mild corneal edema was observed in 8 eyes and there were slight inflammatory response in the anterior chamber of 11 eyes. The corneal edema and inflammatory reaction were eliminated after one week of application of topical steroid drops.

The preoperative and postoperative visual acuity is shown in Table-I. Three months after surgery, the BCVA improved in 29 eyes. The preoperative BCVA was 0.21±0.18 (logMAR 0.67±0.29) and the BCVA of 3 months after surgery was 0.65±0.32 (logMAR 0.23±0.15). The BCVA of three months after surgery improved significantly compared with that before surgery (*Z*=-10.547; *p*<0.05) (Table-II). The preoperative IOP was 14.7±4.0 mmHg and the IOP of three months after surgery was 14.0±3.6 mmHg. There were no significant differences in IOP before and after surgery (*Z*=-0.474; *p*>0.05) (Table-II).

**Table-I:** Preoperative and postoperative BCVA in post-vitrectomy cataract surgery.

LogMAR Visual Acuity (Snellen)	Preop	Postop
0.0–0.1 (20/20–20/25)	0	7
0.2–0.3 (20/32–20/40)	3	10
0.4–0.5 (20/50–20/63)	9	8
0.6–0.7 (20/80–20/100)	11	4
0.8–1.0 (20/125–20/200)	8	2

**Table-II:** Values of the preoperative and postoperative parameters

Parameter	Preop	Postop	p	Z
Best corrected visual acuity (decimal/logMAR)	0.21±0.18/ 0.67±0.29	0.65±0.32/ 0.23±0.15	0.000^*^	-10.547
Intraocular pressure (mmHg)	14.7±4.0	14.0±3.6	0.635^**^	-0.474
Endothelial cell densities (cells/mm^2^ )	2561±162	2341±159	0.000^**^	-3.916

* The Mann-Whitney test was performed to compare the BCVA (logMAR).

** The χ^2^ were performed to compare the other parameters.

The preoperative endothelial cell densities were 2561±162 cells/mm2 and the densities of three months after surgery were 2341±159 cells/mm2. There was a statistically significant postoperative decrease in endothelial cell densities (*Z*=-3.916; *p*<0.05) (Table-II). The mean endothelial cell loss was -8.71%.

**Fig.1:**
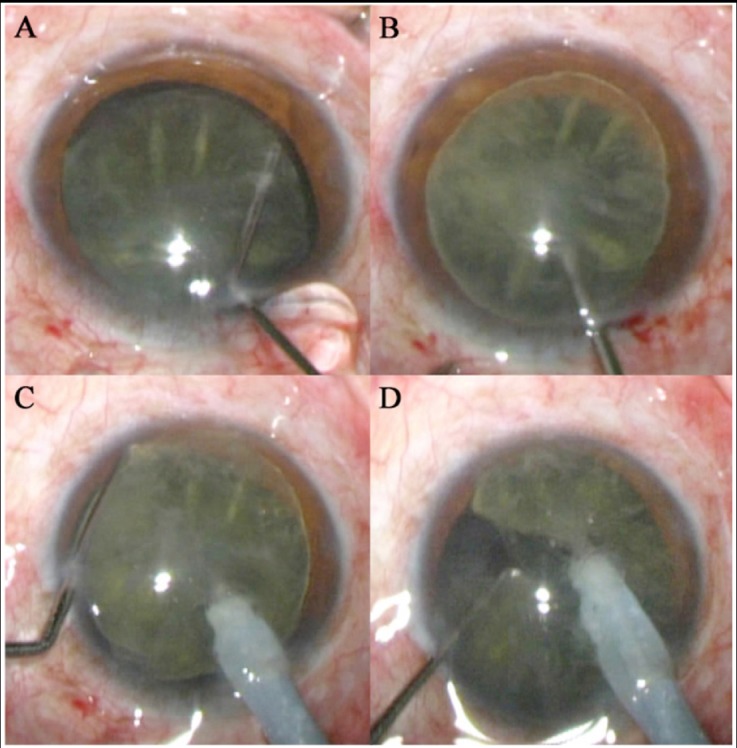
The clinical photographs of surgical steps **A:** Adequate Hydrodissection; **B:** The nucleus was rotated out of the capsule; **C and D:** The nucleus was held firmly by the phaco tip, meanwhile the chopper tip hooks the equator of the nucleus and moves centripetally inward from the periphery.

The posterior capsular opacification were observed in three eyes, two of which were denser, and the YAG laser was performed for posterior capsular incision. There were no corneal endothelial decompensation, IOL decentration or dislocation, secondary glaucoma, endophthalmitis, cystoid macular edema, choroid and retinal detachment, and other ocular complications.

## DISCUSSION

Cataract development is one of the most common complications after vitrectomy.^1^,^7^ In the adult population, the reported incidence of new or progressive lens opacities after vitrectomy ranges from 12.5% to 80% for nuclear sclerotic cataracts and 4% to 34% for posterior subcapsular cataract.^8^,^9^ The risk factors for the development and progression of cataract include intraoperative lens touch, silicone oil or gas injection, intraoperative perfusion and postoperative inflammatory response, etc.^10^,^11^

In order to correct post-vitrectomy cataracts, phacoemulsification combined with intraocular lens implantation is usually performed. However, this surgery has challenging features, which include poor pupillary dilatation caused by the postoperative inflammation, more mobility of the posterior capsule due to the absence of the vitreous, loose zonules and pre-existing posterior capsular dehiscence associated with lengthy vitreoretinal surgery, multiple procedures, and vitrectomy involving vitreous base dissection.^5^,^12^ Thus, the surgery for post-vitrectomy cataract may have more difficulties and higher probability of complications.^13^ In this study, we improved the traditional surgery by performing phacoemulsification outside the capsule to treat the cataract after vitrectomy and achieved good surgical results.

The surgical principles and techniques are as follows: During the process of phacoemulsification, it is important to reduce stress on the zonules and posterior capsule, which are very fragile after vitrectomy.^14^ In addition, small pupils may have a negative effect on the operation in some cases, requiring the surgeon to minimize damage to the intraocular tissue.^15^ The key to surgical technique is that the nucleus was transferred out of the capsular bag and the phacoemulsification was performed in the anterior chamber.

As we know, adequate anterior chamber space must be maintained in the process of operation to reduce the injury of corneal endothelium.^16^ Without vitreous support, as the nucleus is rotated out of the capsule into the anterior chamber, the lens-iris diaphragm move backwards, and the nucleus return back to its original position, thus the anterior chamber depth increases.^14^ Therefore, the intraocular apparatus and ultrasonic energy can maintain a safe distance from the corneal endothelium, without much damage to the corneal endothelium and other intraocular tissues.^17^

Endothelial cell loss is a major concern because a primary complication of cataract surgery is postoperative corneal decompensation.^16^ Studies of diverse phacoemulsification techniques report endothelial cell loss ranging from 5% to as high as 23%.^16^,^18^ Our result showed that there was a statistically significant decrease in endothelial cells after three months. The endothelial cell loss in our study was 8.71%, which is not higher than the findings in previous studies.^16^,^18^ It suggests that phacoemulsification in the anterior chamber does not cause more damage to corneal endothelial cells.

In addition, the nucleus outside of the capsular bag was prone to shift and flip, as it was not fixed by the capsule. Therefore, we adopt the direct phaco-chop technique to deal with the nucleus, which means that the nucleus is held firmly by the phaco tip, meanwhile the chopper tip hooks the equator of the nucleus and moves centripetally inward from the periphery to segment the nucleus.^16^ In this way, chopping applies much less force against the zonules and capsule because the phaco tip secures the nucleus, and the manual instrument forces are directed centripetally against each other.

Notable fluctuation of anterior chamber depth may occur because of increased movement of the lens-iris diaphragm.^9^ We solved the problem by keeping the bottle height low and maintaining irrigation whenever the phaco probe or irrigation-aspiration probes were in the eye.

Previous studies have shown that marked posterior capsular fibrosis or plaques were quite common in silicone oil filled eyes. Centrally located plaques may be visually significant and need to be removed. During our operation, the light plaques were removed by capsular polishing and the very dense plaques were managed by posterior capsulorhexis. The posterior capsular turbidity after surgery was performed by YAG laser incision.

In our study, the BCVA improved in 93.55% eyes after surgery, and no serious complications occurred during and after the operation. This proves that the modified form surgical technique, with phacoemulsification in the anterior chamber, is safe and effective in post-vitrectomy cataracts.

## CONCLUSION

Our study showed that the principles of phacoemulsification in the anterior chamber discussed in this paper would hopefully minimize surgical difficulty and help reduce complications to deal with post-vitrectomy cataracts. Despite a significant decrease in endothelial cells after surgery, the loss of endothelial cells was still in the safe range. In summary, phacoemulsification in the anterior chamber is a safe and effective technique in post-vitrectomy cataracts.

### Author’s Contribution

**TY:** Designed this study, performed surgery and prepared the manuscript.

**XH:** Analyzed data and prepared the manuscript.

**YL and YZ:** Responsible for collecting data and postoperative clinical assessment.
